# 电离辐射降低NSCLC细胞株T790M突变所致TKI耐药

**DOI:** 10.3779/j.issn.1009-3419.2015.08.02

**Published:** 2015-08-20

**Authors:** 静 李, 新虎 武, 振 王, 泽天 沈, 妮 孙, 锡旭 朱

**Affiliations:** 210002 南京，南京大学医学临床学院南京军区南京总医院 Department of Radiation Oncology, Jinling Hospital, Medical School of Nanjing University, Nanjing 210002, China

**Keywords:** 肺肿瘤, 放射敏感性, 表皮生长因子受体酪氨酸激酶抑制剂, 药物耐受性, T790M突变, L858R突变, Lung neoplasms, Radiosensitivity, EGFR-TKI, Drug tolerance, T790M mutation, L858R mutation

## Abstract

**背景与目的:**

以表皮生长因子受体（epidermal growth factor receptor, EGFR）为靶点的分子靶向治疗在非小细胞肺癌（non-small cell lung cancer, NSCLC）的治疗中发挥重要的作用。*EGFR*突变的患者对EGFR酪氨酸激酶抑制剂（EGFR-tyrosine kinase inhibitor, EGFR-TKI）治疗敏感、疗效显著，但无论近期疗效如何，患者最终都不可避免地产生耐药。大量研究证实，*EGFR*基因的二次突变（T790M）是患者耐药的主要原因。本研究旨在探讨电离辐射对NSCLC细胞株T790M突变所致EGFR-TKI耐药的影响。

**方法:**

选择NSCLC细胞株H1975和H3255为研究对象，实时荧光定量PCR法检测两株细胞的突变状态、克隆形成实验观察两株细胞的放射敏感性，MTT法检测各处理组两株细胞对EGFR-TKI的抗药性。

**结果:**

H1975为T790M+L858R双突变株、H3255是仅有L858R的单突变株；各处理组H1975及H3255的存活分数未见明显差异（*P*=0.952），提示T790M突变对NSCLC细胞株的放射敏感性无影响；2.5 Gy X线辐射组，H1975的IC_50_为（0.678, 2±0.373）μmol/L，较0 Gy对照组的（3.520±0.821）μmol/L明显下降，差异有统计学意义（*P*=0.008），H1975相较于H3255的抗药性也从85.9倍下降为39.2倍。

**结论:**

电离辐射可降低NSCLC细胞株T790M突变所致的TKI耐药，本实验的研究结果为后续的体内和临床研究提供了研究依据；EGFR-TKI治疗期间联合放射治疗对克服T790M突变介导的耐药性有望成为一种有希望的治疗策略。

## Abstract (en)

**Background and objective:**

Epidermal growth factor receptor-tyrosine kinase inhibitor (EGFR-TKI), which targets EGFR, plays an important role in non-small cell lung cancer (NSCLC) treatment. Patients with somatic activating mutations in the *EGFR* gene exhibit significant initial response but eventually develop resistance to TKI. The second mutation (T790M) of the *EGFR* gene is the possible main cause of drug resistance. The aim of this study is to investigate the effect of ionizing radiation on EGFR-TKI resistance caused by T790M mutation in NSCLC cell lines.

**Methods:**

We selected H1975 and H3255 as research subjects and tested the mutation states by real-time PCR analysis. Radiosensitivity was determined by clone-forming test, and drug resistance was detected in different groups by MTT assay.

**Results:**

H1975 is an *EGFR* double mutant (L858R plus T790M), whereas H3255 is an *EGFR* single mutant (L858R). The cell survival fractions of H1975 and H3255 did not vary in different treatment groups (*P*=0.952). Thus, T790M mutation did not affect the radiosensitivity of NSCLC cell lines. The IC_50_ of H1975 in the 2.5 Gy group [(0.678; 2±0.373) μmol/L] was statistically significant compared with that in the 0 Gy normal control group [(3.520±0.821) μmol/L] (*P*=0.008). The drug tolerance of the H1975 cell line by 89.5 dropped to 39.2 times.

**Conclusion:**

Ionizing radiation can reduce TKI resistance caused by T790M mutation in NSCLC cell lines. Our results provide a research basis for future *in vivo* and clinical studies. Radiotherapy combined with EGFR-TKI treatment can be a promising strategy to overcome T790M-mediated drug resistance.

以吉非替尼为代表的表皮生长因子受体酪氨酸激酶抑制剂（epidermal growth factor receptor-tyrosine kinase inhibitor, EGFR-TKI）是肺癌治疗的有效方法，尤其对*EGFR*突变的、非吸烟、亚裔女性患者，其疗效尤为显著；遗憾的是随着治疗时间的推移，TKI的耐药不可避免。研究^[1-4]^证实*EGFR*基因的二次突变（T790M）与至少50%的耐药患者相关。目前，EGFR-TKI耐药患者的后续治疗成为亟待解决的重要问题。放射治疗是非小细胞肺癌（non-small cell lung cancer, NSCLC）治疗的重要方法。理论上讲，放射治疗可以迅速消除局部病灶，能够有效降低系统治疗中存在的耐药性；与此同时，越来越多的临床回顾性分析结果^[5-7]^提示：TKI治疗期间进行局部治疗可以降低患者的抗药性，显著延长患者的无进展生存期及总生存期，使患者获益。本研究旨在探讨电离辐射对NSCLC细胞株T790M突变所致耐药的影响，为探索TKI耐药NSCLC患者的治疗提供新的思路。

## 1 材料与方法

### 1.1 细胞株及主要试剂

人肺腺癌H1975、H3255细胞株均购于北京北纳创联生物技术研究院、*EGFR*基因突变检测试剂盒购于厦门艾德生物技术有限公司、吉非替尼（Gefitinib）粉剂购于大连美仑生物技术有限公司、DMEM培养基为美国GIBCO公司产品、DNA提取试剂盒为美国Omega公司产品、MTT粉剂为美国Sigma公司产品、直线加速器为瑞士医科达公司产品、激光共聚焦显微镜为日本Olymupus公司产品、PCR扩增仪ABI7900为美国ABI公司产品、酶标仪Micoplate Reader 450为美国Bio-Rad公司产品。

### 1.2 实时荧光定量PCR

对数生长期的H1975、H3255细胞按DNA提取试剂盒的操作步骤提取细胞DNA，测定其浓度；将提取的DNA用超纯水稀释至终浓度为2 ng/μL；按照*EGFR*基因突变检测试剂盒的操作步骤，加样，上机，PCR反应条件为：第一阶段：95 ℃ 5 min 1个循环；第二阶段：95 ℃ 25 s、64 ℃ 20 s、72 ℃ 20 s，15个循环；第三阶段：93 ℃ 25 s、60 ℃ 35 s、72 ℃ 20 s、31个循环；信号收集：第三阶段60 ℃时收集FAM和HEX（或VIC）信号，执行实时PCR，保存文件；实验重复3次^[8]^。

### 1.3 克隆形成实验

取对数生长期的细胞，按照X线辐射剂量将不同数目的细胞分别接种于60 mm细胞培养皿中（0 Gy、2 Gy、4 Gy、6 Gy、8 Gy每皿接种细胞数分别为300个、600个、2×10^3^个、5×10^3^个、1×10^4^个，每个剂量组设3个平行孔，实验重复3次）；细胞贴壁后用6 MV-X线进行照射，置于37 ℃、5%CO_2_培养箱中继续培养10 d-14 d，定期换液；当肉眼可见细胞克隆时，弃去培养基，PBS冲洗3遍，4%多聚甲醛固定15 min；加入适量的结晶紫染液染色20 min左右，流水冲洗去除染液，室温放置进行干燥；倒置显微镜下计数每皿细胞克隆数（计数≥50个细胞的单克隆）：计算未照射组克隆形成率（plating efficicy, PE）和各个剂量下的细胞存活分数（survival fraction, SF），PE=（0 Gy剂量下形成的克隆数/细胞接种数）×100%，SF=某一剂量照射下细胞形成的克隆数/（该组细胞种植数×PE）。

### 1.4 MTT实验

取对数生长期细胞，调整细胞浓度，按照5, 000个每孔的密度接种于96孔板中，置于37 ℃、5%CO_2_培养箱中培养24 h；细胞贴壁后用6 MV-X线分别按照0 Gy、1 Gy、1.5 Gy、2 Gy、2.5 Gy进行照射；将Gefitinib用二甲基亚砜（DMSO）溶解，进行倍比稀释后每孔中加入设定浓度Gefitinib，稀释至所需要的工作浓度。一般设5个-7个浓度梯度（实验中的浓度分别为：100 μmol/L、10 μmol/L、1 μmol/L、1×10^-1^ μmol/L、1×10^-2^ μmol/L、1×10^-3^ μmol/L、1×10^-4^ μmol/L、1×10^-5^ μmol/L），每孔100 μL，设5个复孔；培养48 h后每孔中加入5 mg/mL的MTT溶液20 μL继续孵育4 h；终止培养，吸去孔内培养基，每孔加150 μL的DMSO置摇床上低速振荡10 min，结晶物充分溶解后，酶标仪（570 nm）测定各孔的吸光度值（A值）；设定调零孔、对照孔；按以下公式计算药物对肿瘤细胞的抑制率：

肿瘤细胞生长抑制率=（1-实验组A值/对照组A值）×100%；将数据处理后，利用Graph Pad Prism 5软件计算IC_50_（半数抑制浓度）^[9]^。

### 1.5 统计学方法

采用Graph Pad Prism 5软件和SPSS 13.0统计分析软件包进行统计学处理，各组实验重复3次，数据取均数，*P* < 0.05为差异有统计学意义。

## 2 结果

### 2.1 H1975、H3255的*EGFR*基因突变状态

采用实时荧光定量PCR法，对实验所用的H3255、H1975细胞株进行*EGFR*基因检测；结果验证实验选择的H1975细胞为L858R+T790M双突变株、H3255细胞为L858R单突变株（图 1）。

**1:**
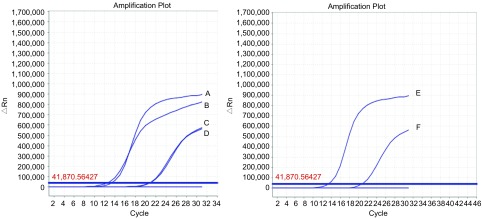
H1975、H3255细胞株的*EGFR*突变检测图（A、E：L858R突变阳性质控曲线；B、D：H1975细胞曲线；C：T790M突变阳性质控曲线；F：H3255细胞曲线）。 The *EGFR* mutation curves of H1975 and H3255 (A, E: positive quality control curves of L858R mutation; B, D: curve of H1975 cell lines; C: positive quality control curves of T790M mutation; F: curve of H3255 cell lines). EGFR: epidermal growth factor receptor.

### 2.2 T790M突变对放射敏感性的影响

克隆形成实验结果显示H1975与H3255细胞的SF2值分别为0.62和0.64。在相同条件处理下，两株细胞生存分数无明显差异（*P*=0.952，图 2，图 3）。结果提示T790M突变对NSCLC细胞株的放射敏性无影响。

**2:**
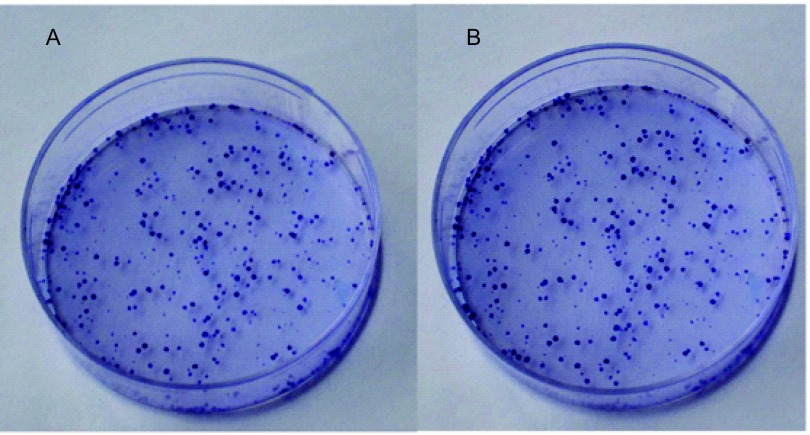
H3255、H1975细胞株的克隆形成情况。A: H3255细胞株；B: H1975细胞株。 The cell clone situation of H3255 and H1975. A: H3255 cell lines; B: H1975 cell lines.

**3:**
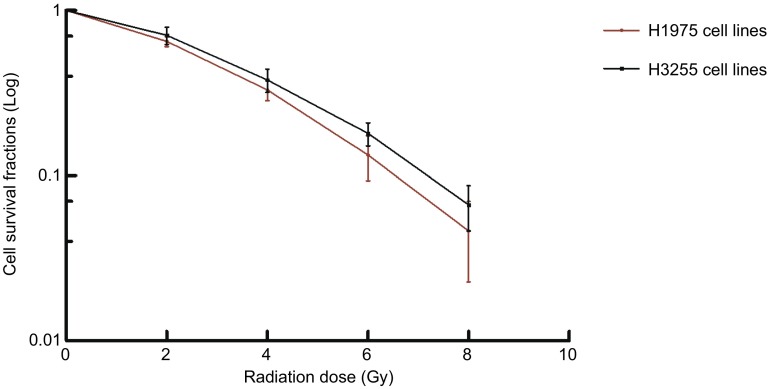
H3255和H1975细胞的剂量-生存曲线图 The dose-survival curve of H3255 and H1975

### 2.3 电离辐射对T790M突变所致吉非替尼耐药的影响

MTT法检测不同剂量X线照射后吉非替尼对H1975、H3255细胞株增殖活性的影响，计算其半数抑制浓度IC_50_。未经X线辐射组H1975的IC_50_为（3.520±0.821）μmol/L，经2.5 Gy X线辐射后降低为（0.678±0.373）μmol/L，差异有统计学意义（*P*=0.008）；未经X线辐射组H3255的IC_50_为（0.041±0.020）μmol/L，经2.5 Gy X线辐射后降低为（0.017±0.009）μmol/L（*P*=0.224）；未进行电离辐射时，H1975的IC_50_是H3255的85.9倍，经2.5Gy X线辐射后降为39.2倍（表 1）。以上结果说明T790M突变是NSCLC细胞耐药的重要因素；电离辐射可降低NSCLC细胞株T790M突变所致的EGFR-TKI耐药性。

**1:** H1975、H3255细胞株不同处理组的IC_50_ The IC_50_ of H1975 and H3255 in different treatment groups

Radiation dose (Gy)	IC_50_ of H1975 (*μ*mol/L)	IC_50_ of H3255 (*μ*mol/L)	a:b^*^
0	3.520±0.821	0.041±0.020	85.9
1	2.685±1.129	0.036±0.013	74.2
1.5	1.939±0.627	0.029±0.007	66.2
2	1.079±0.801	0.022±0.011	48.2
2.5	0.678±0.373	0.017±0.009	39.2
^*^a:b stand for the ratio of IC_50_ measured by A and B. IC_50_: inhibitory concentration 50%.			

## 3 讨论

近年来，对于NSCLC的治疗，化疗的地位虽然未发生根本动摇，但其疗效已趋于平台，较严重的毒副反应也限制了临床应用。靶向治疗因其可靠的疗效且毒性和不良反应轻，已成为最受关注和最有前途的治疗方法之一^[10, 11]^。40%-80%的NSCLC过度表达*EGFR*基因，是肿瘤发生、发展的关键因素，已成为肺癌治疗的关键靶点。接受EGFR-TKI治疗的*EGFR*基因敏感突变的NSCLC患者，通常在9个月-10个月后出现疾病进展，提示出现继发性耐药^[12-16]^。

对于EGFR-TKI耐药后治疗的高级别循证医学证据较少，一系列的相关研究正在进行中。一项回顾性研究探讨了EGFR-TKI治疗出现疾病进展后的治疗模式。根据患者的疾病控制时间、肿瘤负荷演变和临床症状6项将患者分为快速进展（疾病控制≥3个月，与以往评估相比，肿瘤负荷快速增加，增殖评分达到2分）、缓慢进展（疾病控制≥6个月，与以往评估相比，肿瘤负荷轻微增加，症状评分≤1分）和局部进展（疾病控制≥3个月，孤立性颅外进展或颅内进展，症状评分≤1分）三种临床失败模式。结果提示缓慢或局部进展的患者建议继续TKI联合局部治疗^[17]^。放射治疗，不管是单独应用或联合化疗，都在肺癌的治疗中起到不可或缺的作用：早期的病灶中它是一种根治性的治疗方法，而在转移性病灶中它起到姑息性的治疗作用^[18]^。越来越多的临床回顾性分析结果提示，NSCLC患者EGRF-TKI治疗期间对局部病灶或复发病灶进行局部治疗是一种有前景的治疗方式，能够提高TKI的疗效。然而，有关放疗对EGFR-TKI耐药影响的基础研究甚少。

本研究选择NSCLC细胞株H3255、H1975为研究对象，两株细胞均为*EGFR*基因突变株。其中，H1975为L858R+T790M双突变株，H3255为L858R单突变株，利用实时荧光定量PCR再次验证两株细胞的*EGFR*基因突变状态，为下一步的研究奠定基础。

本研究克隆形成实验结果提示相同剂量辐射条件下，两株细胞的生存分数未见明显差异，这说明两株细胞的放射敏感性相同，T790M突变的存在对NSCLC细胞的放射敏感性无影响，这与既往的研究^[19]^结论相同。

利用MTT实验检测两株细胞对吉非替尼的敏感性，结果提示未经X线处理组H1975细胞株的IC_50_是H3255的85.9倍。既往Pao等^[20]^研究结果显示，H1975细胞株对吉非替尼的敏感性比H3255小100倍，本实验结果与Pao等^[20]^研究结果相似，进一步验证了T790M突变是NSCLC患者EGFR-TKI耐药的重要原因。利用6 MV-X线对两株细胞进行不同剂量辐射，观察两株细胞对吉非替尼敏感性的变化，结果显示H1975与H3255的IC_50_随辐射剂量的增大逐渐降低；当辐射剂量为2.5 Gy时，H3255与H1975细胞株的IC_50_之比，由85.9倍降低为39.2倍；说明电离辐射可以降低NSCLC细胞株T790M突变所致TKI的抗药性。电离辐射可使T790M突变所致吉非替尼耐药发生逆转，其机理尚不明确，需要更多更深入的研究，有研究认为，其中一种可能是电离辐射使*EGFR*发生了第三次突变^[21]^；而Gerlinger等^[22]^的观点则认为由于肿瘤细胞存在遗传克隆异质性，电离辐射可使H1975的T790M突变量相对降低。

综上所述，肿瘤的发生发展是多基因、多阶段、多因素的过程，受肿瘤体积、药物渗透性等因素的影响，单纯靶向治疗不能实现肿瘤根治性治疗的目的。靶向治疗和放疗等治疗方法结合是靶向治疗发展的必由之路。本研究结果促使我们进一步研究电离辐射降低TKI耐药的可能机制，为EGFR-TKI耐药患者的治疗提出一种有希望的治疗策略，为以后的综合治疗模式和临床研究提供理论基础。
